# The First Symptoms of Intoxication by Mustard Gas

**Published:** 1918

**Authors:** 


### GAS INTOXICATION

***The First Symptoms of Intoxication by Mustard Gas.*** By A. GIRAUD. Trans. and Abs. from the *Jour. de Méd. et de Chir. Prat.*, Nov. 25 1917.

The author remarks that although the symptoms resulting from mustard gas are all late, the various types of lesion appear at different times.

Lesions of the eye appear first. They are clearly defined within from 6 to 12 hours of the gas attack, and are completely established within from 24 to 48 hours.

Burns, if serious, may develop as early as the conjunctivitis, or even earlier. Burns of a less serious nature, however, may not appear until the 4th or 5th day. Sometimes they are not evident until the 10th or 15th day.

The respiratory lesions appear within from 2 to 4 days.

Eye lesions are the most frequently observed. They are of the congestive type. The vessels of the conjunctive tissue appear sinuous and dilated, and are of a bright red color. Sometimes the confluence of the vessels leads to the forming of little pools the size of a lentil. Although in serious cases the pain may be very great, it is usually not very marked, and in some instances it is wholly lacking. There is very little tear secretion and the sight is rarely impaired. The pupils react to light and distance. The lids may be swollen. Light eye lesions usually heal within from two to three days.

Lesions of the respiratory organs are usually localized to the larynx, the trachea, and the large bronchi. The larynx is especially affected. On the other hand, the intermediary and small bronchi are not affected. The usual bronchitis sounds are not audible. It appears that the gas loses its power as it advances into the respiratory canals.

Aphonia is the first symptom, appearing almost at once, but sometimes preceded by fits of coughing. The aphonia is complete and lasts for four or five days. The coughing is painful and sounds like the whooping cough. At the end of two or three days mucous and purulent matter is coughed up. It becomes difficult to breathe lying down. Hemoptysis has never been observed by the author.

Unlike the lesions of the eye, the respiratory disturbances are very tenacious. Frequently when they appear to be almost well, all the symptoms may break out again. There may be two or three such relapses.

Serious burns appear within 12 hours. A large inflamed area is covered with large blisters filled with serum and sometimes with purulent matter. Sometimes instead of blisters there are small vesiculae enclosing the roots of the hairs. The pain is always great. Late appearing burns are generally insignificant, and are much like sun-burn. They are rarely observed on exposed parts.

Prophylaxis and Treatment. For the conjunctivitis and burns the author finds a solution of bicarbonate of soda (30 per 1,000) helpful as a preventive measure if used immediately after the gas attack, and as a wash for the lesions to be followed by bandaging with Vincent powder (boric acid and hypochlorite of chalk).

The author finds the respiratory lesions resistant to all treatment. He thinks purely symptomatic remedies such as expectorants, counter-irritants, and opiates may help.

